# Global Perspectives in AKI: Argentina

**DOI:** 10.34067/KID.0000000000000140

**Published:** 2023-05-24

**Authors:** Javier De Arteaga, Fernando Lombi, Rafael Avila

**Affiliations:** 1 Hospital Privado Universitario de Córdoba: Hospital Privado Centro Medico de Cordoba, Córdoba, Argentina; 2 Hospital Británico de Buenos Aires: Hospital Britanico de Buenos Aires, Buenos Aires, Argentina; 3 Hospital Jose Maria Cullen, Santa Fe, Argentina

**Keywords:** AKI, angiotensin, cytokines, dialysis, hypotension, kidney, microalbuminuria, mortality, nephropathy, renal function

## Introduction

AKI significantly affects worldwide morbidity, mortality, and health care costs. Similarly, it represents the more frequent cause of consultation of hospitalized patients in the nephrology services.^1^ Like many other developing countries, Argentina is not exempt from this reality, lacking detailed registries assessing this condition. In consequence, there is no clear strategy to face this nonmeasured problem.^2^

Furthermore, the health care system in Argentina is highly biased, and the accessibility to medical attention differs in regions with high incomes (Buenos Aires, Santa Fe, Córdoba, and Mendoza) from those with low incomes (north and south regions of the country). In this article, we intend to analyze the current clinical situation of patients with AKI in different settings and their access to therapy.

## Incidence of AKI

The projected prepandemic incidence of AKI in Argentina would be approximately 357,000 patients/yr. The projected incidence of AKI-requiring kidney support therapy (KST) would be approximately 93,093 patients/yr.^2^ The lack of official data limits reporting the ratio of patients with AKI divided by patients at risk during the observation period.

Recently, the AKI-EPI study,^3^ a worldwide registry, disclosed an incidence of 38% of AKI in the ICU setting in Argentina, which matches the incidence of 31.5% published for South America.

There is a general presumption that AKI is an underdiagnosed medical condition because of a lack of uniformly adopted classification. So, rapid identification is essential to avoid progression toward more severe stages of AKI. Those patients developing AKI-requiring KST show a high mortality rate ranging from 45% to 59%^4–13^.

Unfortunately, no official data exist regarding the proportion of patients with AKI who progress to ESKD; estimations are approximately 12.5%. Furthermore, the causes contributing to the progression from AKI to ESKD are unknown because the patients are not referred to the nephrologist for follow-up.

## Identification and Diagnosis of AKI

Timely recognition is paramount for managing AKI. Our country's diagnosis of AKI is based on Kidney Disease Improving Global Outcomes recommendations, which cannot detect the subclinical and early stages of the disease. Identifying kidney stress or damage through biomarkers is not currently available because of our country's scarcity of assays or tests. In addition, the level of evidence to support their use has yet to be conclusive. Nevertheless, as of October 2022, the biomarker urinary insulin-like growth factor-binding protein and tissue inhibitor of metalloproteinase will be commercially available in Argentina.

## How Does AKI Affect Argentina?

The way AKI affects Argentina is related to the unique features of a vast country (eighth largest in the world with 2.78 million square km^2^). The population distribution pattern shows high-density populated areas with 15.000 inhabitants/km^2^ in Buenos Aires, while others only concentrate on 1.1 inhabitants per km^2^. This heterogeneous density with highly vulnerable areas accounts for the distribution of scarce resources with poor logistics and infrastructure in some regions. The poverty line rounds in 50% of the population.^2,5^

Argentina has the most significant inequality measured through the Gini coefficient (0: perfect equality and 1: perfect inequality), being 0.42 when compared with Latin America 0.52 or Sub-Saharan Africa 0.56.^6^ Therefore, the causes of the AKI are diverse, requiring a single approach given the high heterogeneity of our geography and the unequal economic situation.

## Causes of AKI in Argentina

According to what was mentioned earlier, AKI causes will depend on whether they are community-acquired AKI or in-hospital AKI.^7^

## Community-Acquired AKI

### Urban or High-Income Areas

The patients have a profile like those in developed countries. They are older with multimorbidity and have access to RRT or ICU. Eighty percent (80%) of this population has access to potable water and 56% a sewage service. The leading causes of AKI include sepsis, postoperative AKI, nephrotoxicity, postcontrast AKI, and iatrogenic causes.^8^

### In Suburban or Low-Income Areas

The patient's profile is like those in low/medium developed countries with a vulnerable population where only 11.6% have access to potable water and 2.5% a sewage service. It accounts mainly for younger people with limited access to health medicine.^8^ Causes mainly include sepsis, dehydration, typical hemolytic uremic syndrome, and obstetric complications. The leading causes may be preventable, and therapies are costly.

### In Remote or Rural Areas

AKI happens mainly in the region where the primary attention occurs in sanitary posts devoid of timely delivered therapies. The leading causes are endemic diseases (dengue, paludism, hantavirus, Fiebre hemorrágica Argentina), snake or spider bites, leptospirosis, hemolytic uremic syndrome, drug-induced AKI, and trauma.

### Hospital-Acquired AKI

Non-ICU settings, nephrotoxicity, postoperative AKI, postcontrast AKI, and glomerular diseases are the more recurrent causes of consultation.^7^ There are no available epidemiological data on this situation in Argentina. This subgroup includes patients with multiple comorbidities and reduced renal functional reserve, making them vulnerable to developing AKI.

ICU setting represents a set of patients at high risk of AKI and AKI-requiring KST. The profile of these patients is defined mainly by hemodynamic instability and multiorgan dysfunction. The leading causes are sepsis, septic shock, and high-risk surgery. A significant difference exists in AKI incidence in ICU in Argentina, only 38% in the AKI-EPI study to 1634 patients per million inhabitants in Santa Fe province.

Given the high proportion of our country's young population, the incidence of trauma is meaningfully elevated, with more than 7000 dead per year. However, the government does not have general data on trauma-associated AKI. In a retrospective study of 611 trauma patients (mean age was 37 years) admitted to the ICU at the Cullen hospital, the overall incidence of AKI was 19.4%. Multivariable logistic regression identified AKI as an independent predictor of mortality (OR, 11.6; 95% CI, 4.51 to 29.82) and LOS-ICU. Thirty-six patients (36%) required RRT.^16^ These findings are similar to other studies where trauma-associated AKI was an independent risk factor for mortality and LOS-ICU.

Other circumstances that may explain the evolution of AKI in Argentina include a scarcity of staff with experience in different RRT, limited access to health centers and diagnostic methods, clinical practice guidelines, and a reduced offer for therapy.^2^

To compare this highly heterogeneous picture of the AKI situation, it may be helpful to consider the reference of the three levels of diagnosis of AKI from the AKI 0 by 25 initiative^17^: better access of the population to renal services and health care, to prevent and eventually adopt external assistance for implementing new health programs.

## Coronavirus Disease 2019 and AKI

The pandemic has had devastating results worldwide. The significant effect has been on the country on low income and middle income. Added to the current limited resources existing, economic turmoil was an unprecedented demand for help with appalling results. A prospective, multicenter cohort study was conducted in 63 ICUs in Argentina. Patients with severe pulmonary disease under mechanical ventilation had a mortality rate of 57%. The incidence of AKI was approximately 52%. AKI was an independent predictor of mortality.^14^ In another retrospective study directed in Santa Fe province evaluating the effect of coronavirus disease 2019 on renal health in the public system, between 2020 and 2021, the number of people who had to start chronic dialysis (CD) and nephrology consultation decreased, compared with the trend of previous years. The mortality rate of patients with AKI requiring KST doubled from regular mortality from 41% to 88%.^15^

## Nephrology Training in Argentina

Argentina has 44 programs distributed throughout the country, many of them validated by the Sociedad Argentina de Nefrología. The number of scholarship holders who graduated per year is 59 (the last 5-year average), of which 25 (43%) are of foreign origin, mainly Spanish-speaking from Latin American countries. As part of their training, the programs include ultrasound training, mainly to guide percutaneous procedures such as catheter insertion for hemodialysis and renal biopsy.

Unfortunately, we need to get data on the proportion of graduating fellows who stay in larger cities or move to smaller ones.

Notably, like other regions worldwide, interest/requests in Argentina for nephrology scholarships are declining, generating a crisis in the medium-short term.

## AKI, RRT, and Modalities

Extracorporeal organ support has grown in the last years in our country, despite the significant economic challenges experienced. Although 90% of the KST is performed as intermittent hemodialysis (IHD), there has been considerable advancement in prolonged intermittent KST (PIKST) and continuous KST (CKST) as a modality of initiation of RRT.^10^ Although most of the renal support treatments (IHD, PIKST) performed in the ICU are ordered and managed mainly by nephrologists in compliance with Argentinian Dialysis Law 22.853, which regulates these activities, the approach to this type of patient is performed indeed as a team with critical care medicine specialists.

Still, given the increasing number of patients with multiple organ dysfunction in critical care areas, intensivists have gained a central role in performing CKST, advocating the development of a dedicated ICU-CKST team. Intensivists in Argentina receive training from various sources, including theoretical education provided by different Sociedad Argentina De Terapia Intensiva (SATI) committees, as well as industry workshops offered at congresses or institutions that have acquired these machines. Despite the economic challenges faced in Argentina, the use of extracorporeal technology continues to grow. Both private and public health care systems face challenges with extracorporeal technology, including personnel and program maintenance costs. However, most CKST procedures are performed in public hospitals using the CVVHDF modality with heparin as the primary anticoagulant.

In Argentina, a survey asked institutions about the feasibility of performing CKST. Only 5% responded that they were able to perform this technique. The main reasons for not performing CKST were lacking human resources (51%) and funding (34%).^13^

## Access to RRT

**Figure 1:**
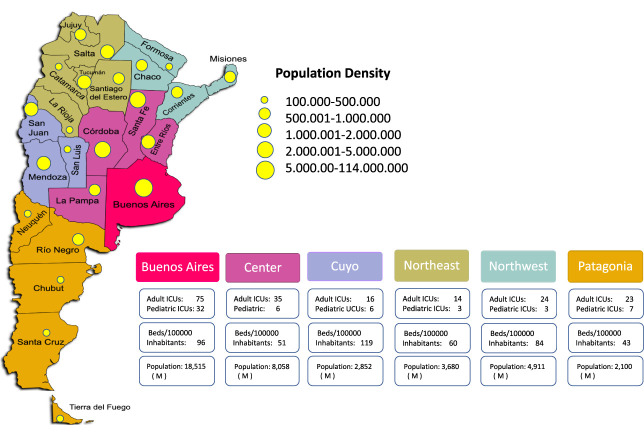
**Numbers of adult and pediatric ICUs, beds/100,000 inhabitants, and population divided per Argentinian regions.** Modified from INDEC. Adapted from Gilardino *et al.*^12^

The Argentine health system guarantees universal access to health care from the tripartite system that includes public health, social security, and the private system.^2^ Theoretically, no patient should be denied dialysis treatment because of financial reasons; reality shows a very different scenario. A self-assessment survey of ICU structure performed by SATI showed a notable concentration of critical care beds with better structural complexity in the wealthier areas of Buenos Aires and the Center regions of the country. Despite their complexity, the availability of these centers to perform RRT 24/7 was lower than 50%^12^ Figure 1.

The current practice regarding the timing of KST in AKI depends on many factors, such as availability of material (disposables), technical and personal resources (training and expertise), and country region (wealthier versus poorer).

This situation opens a debate within our society about why only a privileged part of our society has access to receive KST although the Argentine government guarantees universal health care; by this particular aspect of the care, the AKI would find themselves under the yoke of the nephro-plutocracy (neologism imposed by Richard Sullivan for high-cost cancer treatments^16^ for which only the wealthiest people in a society have access to this type of treatment, establishing this situation as a bioethical dilemma because some principles that govern it are subverted).

During the economic stability in our country, a study investigated the cost-effectiveness ratio (CER) of applying CKST in hemodynamically unstable patients as an initial modality in the Social Services for the Elderly in Argentina. CKST was slightly less expensive and more beneficial than other modalities, with a probability in the CER analysis of 65.4%.^11^

## Upcoming Challenges

The Argentinian health care system, together with the support of scientific societies, should focus on three lines of action to manage patients with AKI (Table 1).

1. To develop a national registry of AKI that will make it possible to learn about the different realities of the country and create a mutual awareness of the importance of completing the registration. If there is no record, there is no way to establish efficiency and equity in the distribution of resources.
2. To foster the training for health personnel in recognition of AKI, giving participation in courses, congresses, scientific meetings, *etc.* Intensifying the training of nurses to increase the efficiency in applying KST in this type of patient will decrease morbidity, mortality, complications, and costs.
3. We must provide robust data to standardize the different modalities of KST. Thus, achieving the recognition by health systems of the other KST modalities, especially PIKST/CKST ones.

**Table 1:** Upcoming challenges

Three lines of action to manage patients with AKI	
Education	• Training in recognizing, preventing, and treating AKI with eclectic approach programs according to the three levels of attention.
Data collection/research	• SATI, SAN, and the government should develop a feasible database. • Determining the different causes of AKI would optimize the different approaches according to a specific region of the country.
Blood purification techniques	• Familiarize both intensivists and nephrologists with new techniques of blood purification. • Take an extracorporeal organ support program as a part of a multidisciplinary approach (machines, institutions, and patients needed and outcomes). • Establish metrics of the KST. • Tandem therapies benefits and risks.

SATI, Sociedad Argentina de Terapia Intensiva; SAN, Sociedad Argentina de Nefrología; KST, kidney support therapy.

In conclusion, the rapid identification of AKI is crucial in improving outcomes. Improving the conditions of access to primary health care centers and adequate care would prevent the development of many acute renal failures. The development of surveillance programs for the early detection of high-risk patients in hospitals should be a standard of care. Argentina has several limitations regarding the diagnosis, access, and performance of KST.

We believe a strategy that includes the development of a registry, training personnel, and standardization of the KST modalities will significantly improve the accessibility of this technique in our country.
